# Acute and Chronic Effects of Dietary Lactose in Adult Rats Are not Explained by Residual Intestinal Lactase Activity

**DOI:** 10.3390/nu7075237

**Published:** 2015-07-08

**Authors:** Bert J. M. van de Heijning, Diane Kegler, Lidewij Schipper, Eline Voogd, Annemarie Oosting, Eline M. van der Beek

**Affiliations:** Danone Nutricia Research, PO Box 80141, 3508 TC Utrecht, The Netherlands; E-Mails: dianekegler@gmail.com (D.K.); lidewij.schipper@danone.com (L.S.); eline.voogd@danone.com (E.V.); annemarie.oosting@danone.com (A.O.); eline.vanderbeek@danone.com (E.M.B.)

**Keywords:** digestion, lactose, lactase, galactosyl xylose, infant milk formula, rats

## Abstract

Neonatal rats have a high intestinal lactase activity, which declines around weaning. Yet, the effects of lactose-containing products are often studied in adult animals. This report is on the residual, post-weaning lactase activity and on the short- and long-term effects of lactose exposure in adult rats. Acutely, the postprandial plasma response to increasing doses of lactose was studied, and chronically, the effects of a 30% lactose diet fed from postnatal (PN) Day 15 onwards were evaluated. Intestinal lactase activity, as assessed both *in vivo* and *in vitro*, was compared between both test methods and diet groups (lactose *vs.* control). A 50%–75% decreased digestive capability towards lactose was observed from weaning into adulthood. Instillation of lactose in adult rats showed disproportionally low increases in plasma glucose levels and did not elicit an insulin response. However, gavages comprising maltodextrin gave rise to significant plasma glucose and insulin responses, indicative of a bias of the adult GI tract to digest glucose polymers. Despite the residual intestinal lactase activity shown, a 30% lactose diet was poorly digested by adult rats: the lactose diet rendered the animals less heavy and virtually devoid of body fat, whereas their cecum tripled in size, suggesting an increased bacterial fermentation. The observed acute and chronic effects of lactose exposure in adult rats cannot be explained by the residual intestinal lactase activity assessed.

## 1. Introduction

The main carbohydrate in milk, the first diet of all mammals, is the disaccharide lactose, which, together with the milk fat fraction, is the main energy source for the growing neonate. The responsible lactose-hydrolyzing enzyme lactase is expressed only in the enterocytes lining the small intestine, comprising the duodenum, jejunum and ileum, and is anchored upon intracellular maturation in the apical, brush border membrane in direct contact with the gut luminal content [1,2].

Most mammals show a temporary and programmed capability to digest lactose: intestinal lactase activity (due to the expression of the lactase gene) is maximal directly after birth and goes down quickly upon weaning [3,4]. This makes sense from both a functional and evolutionary point of view, as milk is normally the only and once-in-a-lifetime source of dietary lactose, the substrate of the lactase enzyme [5].

Yet, it was shown that intestinal lactase gene expression even increased in most mammals toward adulthood, although this expression usually does not result in functional lactase activity, probably due to a reduced or altered post-translational processing of the gene product [2,4,6]. Mammalian lactase gene expression hence seems transitional and shows a disparity with lactase activity or functionality.

Only in man, and even then restricted to specific ethnic groups, a fully functional intestinal lactase activity is maintained after weaning, in line with a continued consumption of lactose, mainly from dairy products [2,4].

In case of insufficient lactase activity, undigested lactose, regarded as “fiber”, enters the large bowel where it can be fermented/metabolized by the residing microflora [7,8]. In this way, the ingested lactose may give some benefit to the organism, although a significant dietary lactose overload may cause gut symptoms due to lactose malabsorption, yielding lactose intolerance. This prompted the current studies into acute and chronic effects of lactose and lactose-containing milk-like products, such as infant milk formula (IMF): What are in adult animals the short- and long-term implications of exposure to lactose? Can adult animals be used to test IMF concepts?

In the current study, first, the time course and extent of the lactose intolerance into adulthood, *i.e.*, the residual lactase activity, was monitored and assessed, both in the absence and continued exposure to dietary lactose (30% lactose diet). To this end, two independent methods to determine the functionality of the lactase enzyme were applied. In addition, the observed residual lactase activity was further studied by monitoring the acute postprandial plasma glucose response to intragastrically-applied lactose loads in adult rats.

## 2. Experimental Section

### 2.1. Animals

Wistar rats from Harlan (Horst, NL) were used, housed in a climate-controlled animal care facility (at 21 ± 2 °C and 50 ± 5% humidity) with a 12/12 L/D cycle with lights on at 5 a.m. Animals were on AIN93-G (American Institute of Nutrition ‘growth’ diet) [9] chow (Teklad Global 18% Protein Rodent Diet, Harlan, Horst, NL) and had free access to food and tap water, unless stated otherwise. All animal procedures were approved by the local Animal Ethics Committee (DEC-Consult, Bilthoven, NL) and were according to laboratory animal care guidelines.

### 2.2. Study A: Intestinal Lactase Activity Assessment

Male and female rat pups born from Wistar dams were employed. Pregnant dams were obtained from Harlan (Horst, NL). On postnatal (PN) Day 2, five nests were culled to 4 pups of each gender per dam. Pups (4 nests) were weaned on PN Day 21 onto an AIN93-G-based chow diet containing 30% lactose (exchanged for maltodextrin) or to the control diet (1 nest) with no lactose. Animals were pair-housed (same gender) and weighed weekly. Diets were available for the pups from PN Day 15 onward. On PN Day 15, 28, 42 and 98 rats from each diet group were given a gavage treatment after a 14-h fast (mainly during the L-period) for *in vivo* lactase activity assessment (see below). The next morning, animals were euthanized by bleeding under inhalation anesthesia (isoflurane). An autopsy was performed, and various organs were collected and weighed. Small intestines were collected for *in vitro* lactase activity assessment (see below).

#### 2.2.1. *In Vivo* Lactase Activity Test

The hydrolysis of the synthetic disaccharide galactose xylose (GX), a suitable substrate for the lactase enzyme, was used to monitor *in vivo* lactase activity. Via gavage, a saline solution was administered containing 4 mg, an adequate amount [10] of the GX-product required (the GX-disaccharide mixture, see below). Upon gavage, animals were put in metabolic cages for 6 h to collect their urine for xylose content (by ion exchange chromatography (IEC)) as a measure for their intestinal lactase activity. The percentage xylose excreted in the urine was assumed to be proportional to the prevailing lactase activity as reported previously [10,11]. Theoretically, the maximal amount to be recovered was 25%: complete GX hydrolysis renders 50% xylose of which only about 50% is excreted by the kidney, the rest being metabolized endogenously [10].

#### 2.2.2. *In Vitro* Lactase Activity Test

The hydrolysis of lactose by a mucosal scraping preparation was used to determine the lactase (EC 3.2.1.23) activity directly with the amount of glucose produced as a read out, according to the method described by Dahlqvist [12]. Excised small intestines (from pylorus to cecum) were thoroughly rinsed with ice-cold saline (0.9% NaCl at 4 °C) to remove luminal contents and were cut open along the longitudinal axis. Mucosal scrapings from the entire small bowel were prepared using glass slides. The collected mucosa was diluted with distilled water (1:5) and homogenized using an Ultra-Turrax blender. The homogenate was kept cold and was spun at ~2000× *g* for 10 min to remove large particles and debris. Next, the lactase activity was assessed in the supernatant: the assay used a lactose solution in maleate buffer (0.1 M, pH 6) to be incubated for 60 min at 37 °C with an equal amount of homogenate (final lactose concentration: 28 mM). Hereupon glucose was assessed colorimetrically using the GOD-PAP (Glucose Oxidase - p-amineophenazone) method (Roche Diagnostics, Almere, NL). Lactase activity was expressed as arbitrary units (U/mL homogenate): 1 unit hydrolyses 1 µmole disaccharide per min. The prepared homogenates contained ~15 mg protein/mL, determined by the BCA assay (Pierce, Fisher Scientific/Emergo, Landsmeer, NL).

### 2.3. Study B: Lactose Testing in Adult Rats

Individually housed male adult rats (initial body weight (BW) 225–250 g) were fitted with a permanent intra-gastric (i.g.) cannula and a jugular vein cannula, both according to local standard procedures [13]. The chronic cannulas allowed for frequent and stress-free i.g. administration of meals and venous blood sampling, respectively, enabling monitoring of the post-meal (postprandial) plasma response upon i.g. meal application. Instilled were carbohydrate solutions (total 2 g/kg BW) or reconstituted infant milk formula (IMF). IMF solutions (Nutrilon^®^, Nutricia, Zoetermeer, NL) contained per 100 mL:1.4 g protein, 3.5 g fat and 7.4 g carbohydrates (>97% lactose). Furthermore, the effect of adding maltodextrin (Glucidex DE19; 0.5 g/kg BW) to IMF was studied.

Animals were fasted for 4 h prior to treatment and received a load of 6 mL/350 g BW via the intragastric cannula, whereupon blood samples were taken. Blood samples (200 µL) were collected in chilled EDTA-coated tubes to avoid coagulation. Plasma was prepared by centrifugation (about 2500× *g* for 15 min at 4 °C) and stored at −80 °C until being assayed. Plasma levels of glucose and insulin were assessed by the GOD-PAP assay (Roche Diagnostics, Almere, NL) and a specific rat insulin ELISA kit (DRG Diagnostics, Veghel, NL), respectively. The insulin ELISA had a detection limit of 22.6 pM; its intra- and inter-assay variabilities were 4.6% and 4.8%, respectively.

### 2.4. GX-Product Preparation

**Figure 1:**
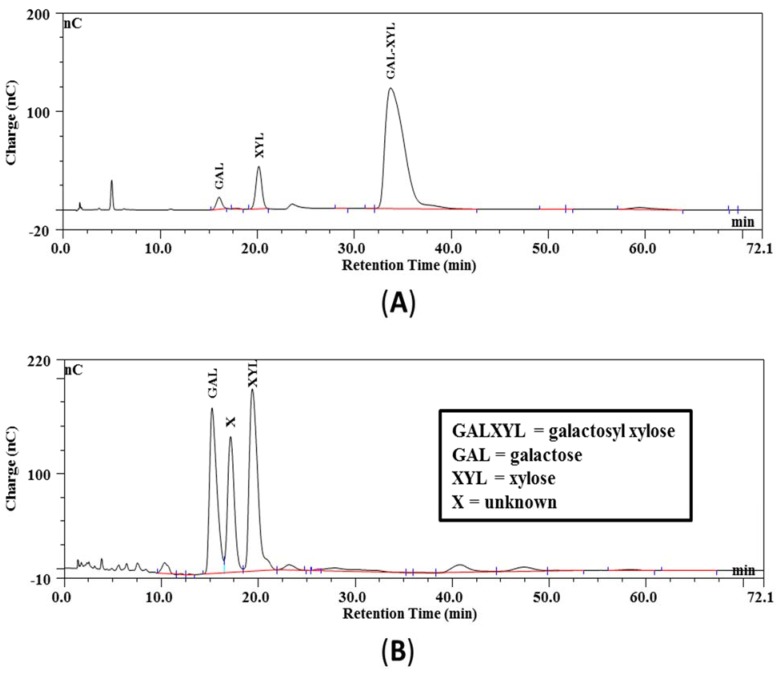
(**A**) Chromatogram of purified galactosyl xylose: before digestion; (**B**) chromatogram of the digestion of the galactosyl xylose into galactose and xylose: after digestion.

The GX product used is not commercially available and, hence, was prepared in our laboratory via enzymatic β-d-galactosylation of xylose, as described by Aragon *et al.* [14] with some modifications. Nitrophenyl-β-d-galactopyranoside (50 mM) and d-xylose (500 mM) were dissolved in warm (37 °C) 0.2 M phosphate buffer (pH 7) to which β-galactosidase from *E. coli* (312 U) was added (all chemicals from Sigma-Aldrich Chemie, Zwijndrecht, NL). After incubation for 22 h at 37 °C, the synthetized disaccharides (2-, 3- and 4-galactosyl xylose, GX) were purified from the crude reaction mixture: initially, filtration removed nitrophenol, and its derivatives were adsorbed to added charcoal. Acetonitrile was added subsequently to the filtrate up to 2%, and this mixture was poured onto a bed of 200 g charcoal (in a Büchner funnel) that was activated earlier by treatment with 0.1% TFA in 80% acetonitrile (“activated charcoal”). After a wash step with 1 L 2% acetonitrile to remove xylose, the disaccharides adsorbed to the activated charcoal bed were eluted in the fourth elution step (100 mL each) with 25% acetonitrile. Evaporation rendered a product that consisted of 95 m/m% GX-mixture and only 2.2 m/m% xylose. Figure 1A shows the IEC chromatogram of the obtained purified GX-product. No attempts were made to further purify the co-eluting disaccharide mixture (2-, 3- and 4-galactosyl xylose, GX), as all three region isomers were good lactase substrates [10]. The GX-product mixture was shown to be entirely digested when incubated with lactase enzyme (see Figure 1B), rendering galactose, xylose and some unidentified digestion product (possibly from the enzyme solution).

### 2.5. Chromatography

GX-product evaluation and urinary xylose content was assessed by ion exchange chromatography (IEC) using a BioLC system (Dionex, Amsterdam, NL). Chromatography employed an isocratic run (72 min) with 15 mM NaOH as the eluent (1 mL/min) and used a Carbopac PA1 guard column (50 × 4 mm) in series with a Carbopac PA1 analytical column (250 × 4 mm). An ED40 detector (Au, 0.015 inch gasket) with a quadric-pulse analyzed the eluate composition. Every five runs, the column was rinsed with 330 mM NaAc followed by 300 mM NaOH, whereupon it was re-equilibrated to 15 mM NaOH again.

### 2.6. Statistics

Data are presented as the means ± SEM. Statistical analysis was performed using SPSS 15.0 (SPSS Benelux, Gorinchem, NL). The effect of treatment was tested using a repeated measures or univariate ANOVA, followed by LSD *post hoc* analysis. Correlations were evaluated by Pearson’s test. Differences were considered significant at *p* < 0.05.

## 3. Results

### 3.1. Effect of Lactose Containing Diets during Development

#### 3.1.1. Study A: Growth

Feeding rats from weaning until PN Day 98 a diet with 30% lactose did not appear to affect body growth or body weight course (growth velocity) into adulthood: the animals on the lactose diet grew well and gained weight within the normal range of Wistar rats, although body weight was on average lower than that observed in the control group. At autopsy on PN Day 98, notably smaller fat deposition was observed compared to controls: virtually no abdominal or subcutaneous fat pads were observed. Furthermore, organ weights were affected: muscle and liver weights were decreased, whereas pancreas and cecum weight was increased in lactose-fed *vs*. control rats (Table 1).

**Table 1:** Body weight and organ weight (in g) of male rats at autopsy on postnatal (PN) Day 98. Data are means (SEM) *n* = 1–2 for controls and *n* = 3 for the lactose diet group. * *p* < 0.05.

Diet	Body Weight	Liver	Pancreas	m. tibialis	Caecum
control	430 (20.0)	15.4	0.92 (0.06)	0.80	3.9 (0.41)
lactose	362 (7.6)	14.0 (0.5)	1.38 (0.02) *	0.73 (0.02)	11.6 (0.83) *

#### 3.1.2. Study A: Intestinal Lactase Activity Assessments

**Figure 2:**
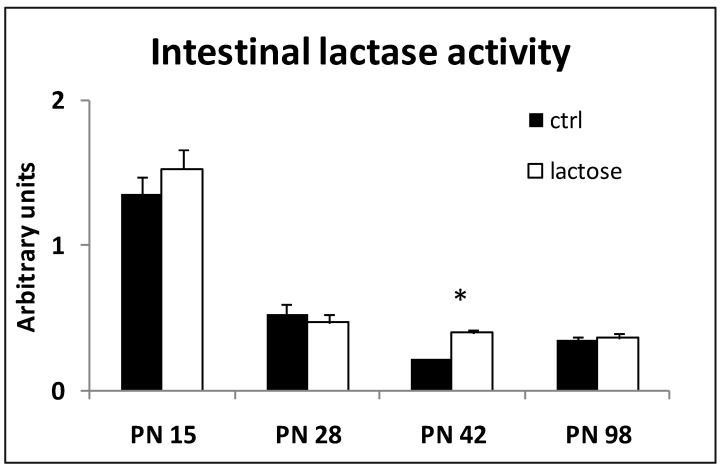
Postnatal (PN) intestinal lactase activity (in U/mL) as determined from mucosal scrapings at various time points; control (*n* = 2), lactose (*n* = 7–8) at each time point; * *p* < 0.05.

Intestinal lactase activity, as assessed in mucosal scrapings, decreased promptly upon weaning (PN Day 21) and remained at a low residual level (25%) toward adulthood (Figure 2). The calculated lactase activity at PN Day 15 was about 100 mU/mg protein and about 25 mU/mg protein on PN Day 98.

**Figure 3:**
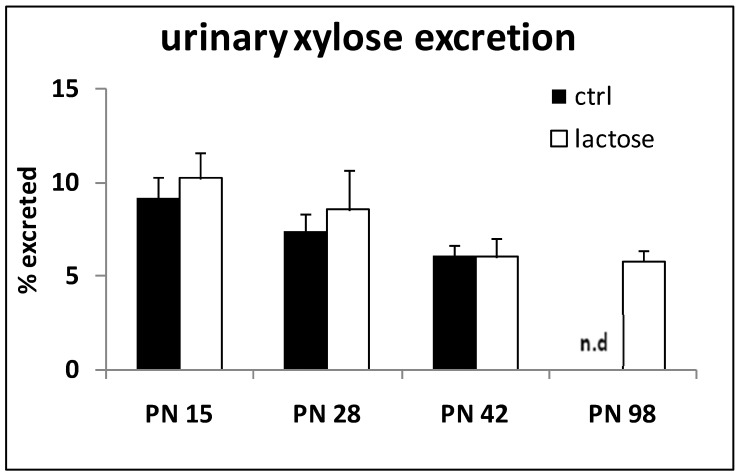
Urinary xylose output over 6 h expressed as a percentage of the amount orally instilled at *t* = 0, assessed at various time points postnatally (PN); ctrl (*n* = 2), lactose (*n* = 7–8) at each time point; n.d.: no data.

Continued exposure to a lactose-containing diet did not alter the observed lactase activity. On PN Day 42, the lactase activity determined in the rats on the lactose diet was found to be statistically different from the controls. Oral application of the disaccharide GX to monitor lactase activity *in vivo* neither differed in time nor between diet groups (Figure 3). Control data on PN Day 98 were not available (no data: n.d.). The maximal urinary xylose recovery found was about 10% in 15-day-old, milk suckling pups. According to this method, the residual lactase activity appears to be maintained at 50% into adulthood.

The urinary xylose excretion did not align with the direct measurement of the intestinal lactase activity: urinary xylose levels did not show a clear fall after weaning as observed with the mucosal scrapings method. Correlation analysis, however, revealed a weak, but significant positive correlation between the two methods used (*r* = 0.41, *p* < 0.05).

### 3.2. Study B: Lactose Testing in Adult Rats

**Figure 4:**
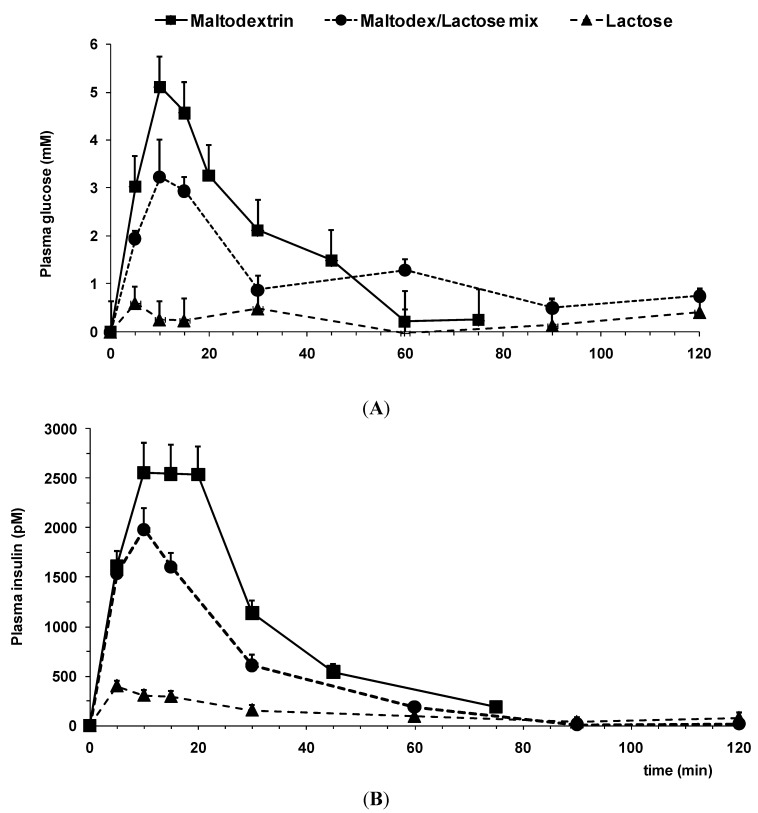
Incremental postprandial plasma glucose (**A**) and insulin (**B**) levels in response to administration of various aqueous carbohydrate loads (*n* = 12–14). Doses instilled (per animal): maltodextrin 0.7 g; lactose 0.45 g; mix: 0.45 g lactose + 0.175 g maltodextrin. Maltodex, maltodextrin.

**Figure 5:**
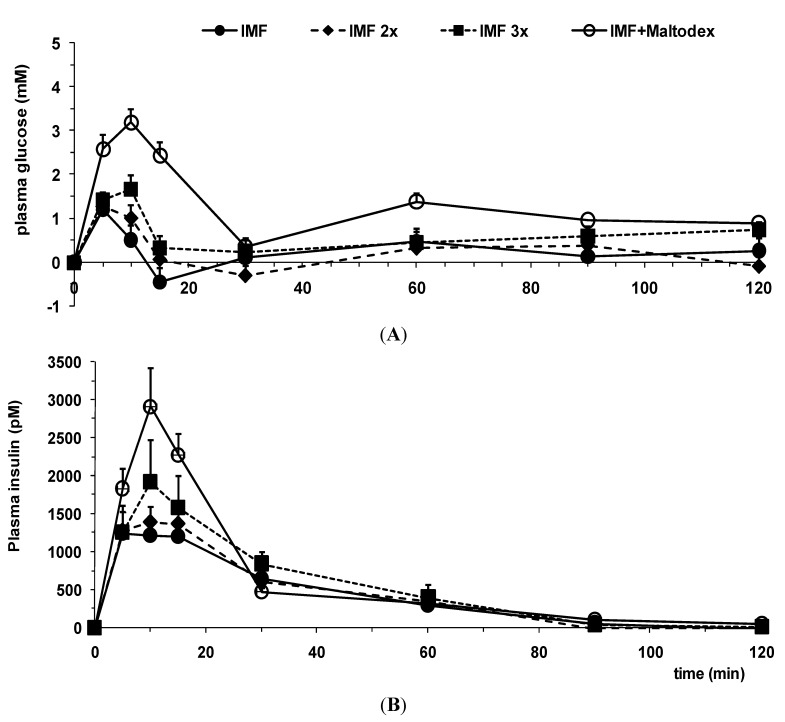
Incremental postprandial plasma glucose (**A**) and insulin (**B**) levels in response to administration of various aqueous IMF loads (*n* = 7–12). IMF, infant milk formula.

The postprandial responses of i.g. instilled loads (6 mL/350 g BW) of aqueous carbohydrate (mix) solutions in adult rats are depicted in Figure 4A,B. Maltodextrin, an easily digestible glucose polymer, administered i.g. in a total dose of 2 g/kg BW, *i.e.*, about 0.7 g per animal, showed a quick and significant response in both glucose (Figure 4A) and insulin (Figure 4B) plasma levels. The infant milk formula (IMF) used in the present tests (Figure 5) contains 7.4 g lactose per 100 mL, *i.e.*, 0.45 g lactose in a 6-mL load. The result of this (albeit lower) carbohydrate load on the plasma glucose and insulin response as shown in Figure 4 is marginal and not statistically different from baseline variability.

However, the addition of only 25% of the above-mentioned maltodextrin dose (0.175 g per animal) to the lactose dose used (as present in IMF) resulted in a significant post-meal rise in plasma glucose and insulin levels (Figure 4).

Hence, in case the carbohydrate load comprises lactose only, the postprandial glucose and insulin plasma response is minimal and much lower compared to carbohydrate loads composed of pure or a blend containing maltodextrin.

Next, IMF was tested: a mixed nutrient solution containing lipids, proteins and carbohydrates; and the postprandial response was compared to instillation of carbohydrates only (Figure 4 *vs.* Figure 5). The presence of macronutrients other than carbohydrates highly affected the postprandial response. In particular, the postprandial plasma insulin response was markedly increased (Figure 5B) compared to the plasma glucose response observed (Figure 5A). Post-meal plasma glucose levels are twice as high, whereas plasma insulin levels even triple (*cf.* the lactose curve in Figure 4 with the IMF curve in Figure 5). Increasing the lactose load by preparing more concentrated IMF solutions (“IMF 2x” and “IMF 3x”) did not significantly affect the height of the postprandial plasma response in glucose or insulin (Figure 5). Thus, tripling the energy and macronutrient density in a nutritive solution containing only lactose as a carbohydrate failed to enhance the postprandial glucose and insulin responses any further.

Again, addition of a small amount of maltodextrin (0.5 mg/kg, *i.e.*, ~0.2 g per animal) to an IMF solution (“IMF + Maltodex” in Figure 5) did cause post-meal plasma levels to rise significantly.

## 4. Discussion

We confirm and observed a major decline in lactase activity during development, although not to the extent as found previously, *i.e.*, to 5%–10% of its original level [1,15,16]. Hence, residual lactase activity was shown to be present in adulthood and was confirmed to be functional by a small rise in plasma glucose levels upon lactose intake, as also shown by others [4,17,18].

As shown previously in artificially-reared rat pups, the ingestion of natural milk seems to play a cardinal role in the maintenance of lactase activity during the suckling period [19], the lactose content in milk not being the most essential component in this respect, nor the only substrate for lactase present in milk [5].

Whether lactase activity levels are linked to lactose-containing milk consumption (as a natural source of lactose) remains controversial [15], despite studies supporting the adaptive theory (or induction hypothesis), stating that keeping the substrate available by continuation of milk ingestion, newborn lactase levels are maintained [20]. We and others [4,16] found no data to corroborate this theory and showed the residual lactase activity in adult rats to be mainly “non-functional” and to elicit merely a small, insignificant post-meal glucose plasma response after lactose ingestion.

The lactase activity in rats was monitored into adulthood by two independent methods: in addition to a direct *in vitro* lactase assay using tissue samples, an indirect, non-invasive *in vivo* method was applied. The activity derived from mucosal scrapings, the average over the entire small intestine (and ignoring possible “hot spots”) showed ~25% of the lactase activity to be retained into adulthood (Figure 2), whereas the adult urinary xylose excretion remained >50% of pre-weaning levels (Figure 3). Furthermore, the mucosal tissue assessment showed an abrupt decrease, almost as an “on/off switch”, a feature not mirrored at all by the *in vivo* method. The methods were previously shown to correlate well and to only differ in their kinetic parameters [10,11]. In all, despite the discrepancies found, both methods do indicate and confirm a considerable residual intestinal lactase activity to be present in adult rats, both in the absence and continued exposure to dietary lactose (30% lactose diet), as also reported previously [15,16,21]. Weaning studies in rats earlier showed the switch from milk to solid diet to occur gradually between postnatal Day 18 and 30, suggesting the decline of functional lactase activity in the course of natural weaning to be gradual, as well [22], and more in line with our *in vivo* data (Figure 3).

Instillation of increasing doses of lactose (0.45 g to 1.35 g as part of a load of 6 mL of IMF solution) yielded only a minor, insignificant rise in post-meal plasma glucose levels (Figure 5A). Moreover, the addition of maltodextrin (25–35 w%) to a lactose load already induced a significant rise in postprandial plasma glucose levels (Figure 4A and Figure 5A). These data show the preference and aptitude of the adult gut to digest and absorb complex glucose-based polysaccharides, such as maltodextrin. In contrast, lactose, although (potentially) digested by the residual lactase activity, apparently is neither efficiently nor rapidly absorbed, even when presented in a higher dosage, to result in significantly elevated plasma glucose levels. Furthermore, insulin levels show a skewed responsiveness to glucose-based carbohydrates compared to lactose (Figure 4B), even when lactose is given mixed with other macronutrients, which also elicit insulin release (Figure 5B).

Two issues deserve to be addressed in this context: Firstly, the glycemic index (GI) of the two carbohydrates is compared. Maltodextrin (in man) is a “fast sugar” (GI 100), whereas lactose is “slow” (GI 45). This already might explain part of the differences in the plasma responses observed. Secondly, the more concentrated IMF solutions instilled (Figure 5) have a higher energy density and osmolality, which might both slow down the gastric emptying rate, adding to the GI effect, although the insulin response seems not affected, indicative of a similar gastric residence time.

The apparent non-functionality of the observed residual intestinal lactase activity was also confirmed in the chronic lactose feeding experiment: despite the low number of animals autopsied (see Table 1), the adult body and organ weight of animals fed a 30% lactose-containing diet indicated that the lactose was not available to the animals as a direct energy source, but as an indirect one (see below). Due to this energy restriction, the animals had less energy to store or to deposit as fat in their adipose tissue, which may explain the lack of fat at autopsy, as also reported previously [23,24]. Alternatively, an involvement of calcium absorption from the gut, which is promoted by lactose [18,24,25], is possible, as high prevailing calcium levels would counteract fat storage [26]. We did not assess calcium levels, nor did we evaluate if the animals on the lactose diet showed compensatory eating. 

In line with the virtual absence of fat, an expansion of the cecal gut compartment in the animals on the lactose diet was observed (Table 1). This indicates that part of the indigestible dietary lactose was treated as a “fiber” and was fermented likewise by the cecal and/or colonic microflora, yielding some, but much less (indirect), energy to the rats. Previously, it was estimated that about 43% of the lactose ingested passes into the colon [8]. As already known [7], adult rats can readily adapt to a high lactose intake without any clinical disorders: indeed, we did not observe loose stools or diarrhea as manifestations of abdominal discomfort or lactose-intolerance in the lactose diet group during the period studied (*i.e.*, PN 15–98). Table 1 also shows liver and muscle weight to be similar (the protein content of the diets was equal), but mentions a bigger pancreas in the lactose diet group. This might possibly refer to the exocrine pancreas trying to boost digestive efficiency by a higher enzyme output.

Lactose-containing weaning diets did not preserve intestinal lactase activity in our rats, despite the adaptive theory claiming this [20]. To date, only peroral gene therapy [17] has been shown in rats to “cure” functional lactose malabsorption permanently through a persistent expression of a β-galactosidase transgene in gut epithelial cells. Treated rats showed no weight loss after two weeks on a lactose-only rat chow and showed a significant post-meal response in plasma glucose levels, both at seven days and at 120 days after gene therapy.

As is known, in man, early postnatal diet impacts the development and body composition in childhood and later life [19,27,28,29]. Nutritional intake during early age (up to four years) may be critical in this respect as a transition from a relatively high fat intake to a relatively high carbohydrate intake occurs at this time [22,29]. As has been shown by observational studies, a higher carbohydrate intake, implying a higher dietary glycemic load (GL), promotes the development of obesity [30]. In this respect, the quality of the carbohydrates ingested seems vital, but to date, no prospective evidence exists to substantiate a pivotal role for carbohydrate quality in early childhood [29]. In addition, dietary factors, including caloric intake, but also a higher meal frequency, resulting in lower postprandial glucose and insulin plasma levels, have been reported to affect body composition beneficially [29]. To address the issue of carbohydrate quality in the early diet, we compared the effect of various carbohydrate loads comprising either maltodextrin or lactose or its combination on the postprandial plasma glucose and insulin response and kinetics in adult rats. Next, whole meals (IMFs) containing lactose and/or maltodextrin were tested and demonstrated the “bias” of the adult GI tract towards the digestion of glucose polymers. Based on these results, we conclude that lactose-containing feeds, e.g., new IMF concepts, should not be tested in adult animals. Similarly, (pre-)clinical studies involving lactose-containing solutions or nutritive preparations, employing adult rats, or animals in general (or even adult men), is therefore cautioned, due to the adult, entirely glucose-based carbohydrate-directed digestive system. Conversely, sucrose or glucose, regularly added to baby and toddler nutrition as an inexpensive lactose alternative, may have detrimental effects on the maturation of the neonatal gut [31,32]. More preferably, human milk replacers (bottle feeds in general) and products designed and produced by the food industry for neonates and/or premature infants [28] should ideally only contain lactose as its major carbohydrate source.

## 5. Conclusions

In conclusion, lactose digestive capacity is transiently present in the rat and declines steeply after weaning. The residual lactase activity shown to be present in adult rats does not directly predict the capacity to digest and absorb lactose-containing food preparations. These findings should be taken into account when studying lactose-containing products in preclinical adult (rat) models.

## References

[1] Kretchmer N. (1989). Expression of lactase during development. Am. J. Hum. Genet..

[2] Witte J., Lloyd M., Lorenzsonn V., Korsmo H., Olson W. (1990). The biosynthetic basis of adult lactase deficiency. J. Clin. Investig..

[3] Sebastio G., Villa M., Satorio R., Guzetta V., Poggi V., Auricchio S., Boll W., Mantei N., Semenza G. (1989). Control of lactase in human adult-tpe hypolactasia and in weaning rabbits and rats. Am.J. Hum.Genet..

[4] Montgomery R.K., Büller H.A., Rings E.H.H.M., Grand R.J. (1991). Lactose intolerance and the genetic regulation of intestinal lactase-phlorizin hydrolase. FASEB J..

[5] O’Connor T.P., Diamond J. (1999). Ontogeny of intestinal safety factors: Lactase capacities and loactose loads. Am. J. Physiol..

[6] Motohashi Y., Fukushima A., Kondo T., Sakuma K. (1997). Lactase decline in weaning rats is regulated at the transcriptional level and not caused by termination of milk ingestion. J. Nutr..

[7] Lawrence J.V., Fischer J.E., Sutton T.S., Weiser H.H. (1956). Adaptation of the rat to a higher lactose diet, effect of the size of the cecum. Ohio J. Sci..

[8] Kim K.I., Benevenga N.J., Grummer R.H. (1978). Estimation of the fraction of the lactose in a high lactose diet available for fermentation in the cecum and colon of the rat. J. Nutr..

[9] Reeves P.G., Nielsen F.H., Fahey G.C. (1993). Ain-93 purified diets for laboratory rodents: Final report of the american institute of nutrition ad hoc writing committee on the reformulation of the ain-76a rodent diet. J. Nutr..

[10] Hermida C., Corrales G., Martinez-Costa O.H., Fernandez-Mayoralas A., Aragon J.J. (2006). Noninvasive evaluation of intestinal lactase with 4-galactosylxylose: Comparison with 3- and 2-galactosylxylose and optimization of the method in rats. Clin. Chem..

[11] Aragon J.J., Fernandez-Mayoralas A., Jimenez-Barbero J., Martin-Lomas M., Rivera-Sagredo A., Villanueva D. (1992). Evaluation of rat intestinal lactase *in vivo* with 4-galactosylxylose. Clin. Chim. Acta.

[12] Dahlqvist A. (1968). Assay of intestinal disaccharidases. Anal. Biochem..

[13] Severijnen C., Abrahamse E., van der Beek E.M., Buco A., van de Heijning B.J., van Laere K., Bouritius H. (2007). Sterilization in a liquid of a specific starch makes it slowly digestible *in vitro* and low glycemic in rats. J. Nutr..

[14] Aragon J.J., Canada F.J., Fernandez-Mayoralas A., Lopez R., Martin-Lomas M., Villanueva D. (1996). A direct enzymatic synthesis of b-d-galactopyranosyl-d-xylopyranosides and their use to evaluate rat intestinal lactase activity *in vivo*. Carbohydr. Res..

[15] Leichter J. (1973). Effect of dietary lactose on intestinal lactase activity in young rats. J. Nutr..

[16] Tadesse K. (1990). The effect of continued feeding of physiological amounts of lactose on the level of intestinal lactase and other disaccharidase enzyme activities in the rat. Exp. Physiol..

[17] During M.J., Xu R., Young D., Kaplitt M.G., Sherwin R.S., Leone P. (1998). Peroral gene therapy of lactose intolerance using an adeno-associated virus vector. Nat. Med..

[18] Abrams S.A., Griffin I.J., Davila P.M. (2002). Calcium and zinc absorption from lactose-containing and lactose-free infant formulas. Am. J. Clin. Nutr..

[19] Yeh K.Y., Holt P.R. (1985). Rat milk maintains intestinal lactase activity in rat pups whereas artificial formulas do not. Pediatr. Res..

[20] Jones D.P., Sosa F.R., Skromak E. (1972). Effects of sucrose, glucose and lactose on intestinal disaccharidases in the rat. J. Lab. Clin. Med..

[21] Jang I., Jung K., Cho J. (2000). Influence of age on duodenal brush border membrane and specific activities of brush border membrane enzymes in wistar rats. Exp. Anim..

[22] Redman R.S., Sweney L.R. (1976). Change in diet and pattersn of feeding activity of developing rats. J. Nutr..

[23] Tomarelli R.M., Hartz R., Bernhart F.W. (1960). The effect of lactose feeding on the body fat of the rat. J. Nutr..

[24] Goseki-Sone M., Maruyama R., Sogabe N., Hosoi T. (2007). Effects of dietary lactose on long-term high-fat-diet-induced obesity in rats. Obesity (Silver Spring).

[25] Ziegler E.E., Fomon S.J. (1983). Lactose enhances mineral absorption in infancy. J. Pediatr. Gastroenterol. Nutr..

[26] Zemel M.B., Miller S.L. (2004). Dietary calcium and dairy modulation of adiposity and obesity risk. Nutr. Rev..

[27] Gall D.G., Chung M., O’Loughlin E.V., Zahavi I., Opleta K. (1987). Effects of parenteral and enteral nutrition on postnatal development of the small intestine and pancreas in the rabbit. Biol. Neonate.

[28] Sangild P.T., Petersen Y.M., Schmidt M., Elnif J., Petersen T.K., Buddington R.K., Greisen G., Michaelsen K.F., Burrin D.G. (2002). Preterm birth affects the intestinal response to parenteral and enteral nutrition in newborn pigs. J. Nutr..

[29] Buyken A.E., Cheng G., Guenther A.L.B., Liese A.D., Remer T., Karaolis-Danckert N. (2008). Relation of dietary glycemic index, glycemic load, addes sugar intake, or fiber intake to the development of body composition between ages 2 and 7 years. Am. J. Clin. Nutr..

[30] McMillan-Price J., Petocz P., Atkinson F., O'Neill K., Samman S., Steinbeck K., Caterson I., Brand-Miller J. (2006). Comparison of 4 diets of varying glycemic load on weight loss and cardiovascular risk reduction in overweight and obese young adults: A randomized controlled trial. Arch. Intern. Med..

[31] Fosbrooke A.D., Wharton B.A. (1975). “Added lactose” and “added sucrose” cow’s milk formulae in nutrition of low birthweight babies. Arch. Dis. Child..

[32] Goda T., Bustamante S., Koldovsky O. (1985). Dietary regulation of intestinal lactase and sucrase in adult rats: Quantitative comparison of effect of lactose and sucrose. J. Pediatr. Gastroenterol. Nutr..

